# A comparison between in vitro and randomized in situ models for remineralization of artificial enamel lesions

**DOI:** 10.1038/s41598-024-76387-w

**Published:** 2024-10-25

**Authors:** Anahita Jablonski-Momeni, Jenna Lentz, Boris Jablonski, Andreas Kiesow, Maria Morawietz

**Affiliations:** 1 Department of Orthodontics, Medical Faculty, Dental School, Philipps-University Marburg, Marburg, Germany; 2 Dental Practice, Lollar, Germany; 3 Fraunhofer Institute for Microstructure of Materials and Systems IMWS, Halle (Saale), Germany

**Keywords:** Enamel, Demineralization, Remineralization, Dentifrice, Fluoride, In-situ, In-vitro, Micro-ct, Dentistry, Oral diseases

## Abstract

The randomized study aimed to evaluate the comparability of in situ (iS) and in vitro (iV) study protocols regarding remineralization of artificial enamel lesions. Two toothpastes (group A: 1450 ppm sodium fluoride, group B: placebo 0 ppm F-), were investigated. IV, a pH-cycling model with toothpaste slurry treatment was applied for 10d. IS, remineralization was performed in 9 participants wearing splints with embedded enamel samples for 10 and 21d, randomly allocated to groups A and B. Samples were scanned by X-ray micro-computed tomography (μCT) and grayscale value line profiles corresponding to mineral density (rel.ΔZ) were analyzed. Statistical analyses were performed using MedCalc Statistical Software, v22.021. T-Test for dependent and independent data and analysis of variance (ANOVA) were used for further analyses (α = 0.05). Rel.ΔZ of fluoride treated samples (A) were iV = 40.2%, iS 10d = 11.5% and iS 21d = 46.1% (p > 0.05). Rel.ΔZ of placebo treated samples (B) were: iV = − 6.2%, iS 10d = 25.2% and iS 21d = 11.0% (p > 0.05). Remineralization potential of both toothpastes was significantly different regarding iV (p < 0.001) and iS after 21d (p = 0.034), while in case of iS 10d no significant difference was detected (p = 0.4). Despite different study protocols the μCT results after remineralization were comparable between iV and iS. The results suggest that selected studies can be carried out in faster, simplified iV studies using pH-cycling instead of iS studies.

## Introduction

The latest Global Burden of Disease study^1^ has shown that caries on permanent teeth affects 2.03 billion people and is the most common disease worldwide. Brushing teeth, ideally with fluoride toothpaste, is the most common form of caries prevention^2,3^. The caries-preventive effect of fluorides is achieved by promoting remineralization, inhibiting demineralization and interfering with bacterial metabolism and plaque formation^4–6^. The efficacy was confirmed in many in situ (iS) and in vitro (iV) studies^7,8^.

In dentistry, remineralization procedures and product efficacy regarding caries prevention and remineralization are usually first investigated in the laboratory (iV) before clinical studies are carried out. Traditionally, these studies evaluate the remineralizing effect of fluoride-containing products (e.g., toothpastes/varnishes/gels) in various compounds or concentrations^9–11^. Laboratory-based de- and remineralization is carried out under standardized conditions, allowing results to be obtained in a short period. Individual behavior patterns and thus a large variability factor in clinical studies are excluded.

As an intermediate step between iV studies and clinical trials, iS studies are proposed to make a balance of merits and limitations between in vitro and in vivo studies and can be used to study the interaction of anti-caries agents and environments^12^. The nature of such models is that tooth slabs are carried in mouth and as such are exposed to de- and remineralization cycles. There are various protocols for performing iS studies to assess remineralisation of the enamel surface. In such studies different types of appliances are suggested, like fixed or removable appliances. Though, the application of removable appliances is more convenient for participants and oral care measurements are easier to perform.

IV and iS models were introduced as an attempt to overcome the challenges that hinder in-vivo studies to provide accurate reflection of what happens intraorally. The major advantages of both methods are the implementation of standard study protocol and different variables can be clearly examined. Moreover, the presence of saliva or oral biofilm adds an advantage of iS model over iV one regarding accurate reflection of what happens intraorally in re- demineralization process.

In a recent systematic review^13^, a large number of in situ studies were included that confirm the remineralizing effect of a fluoride-containing toothpaste in both children and adults. A toothpaste with 1500 ppm fluoride has a better preventive effect with daily use than a toothpaste with 1000 ppm, especially in young permanent teeth^14^. In all cases^13^, a fluoride-free toothpaste was used for the placebo group to confirm the hypothesis aimed for in each study.

In addition to the iS studies mentioned above, there are numerous iV studies on fluoride-assisted remineralization. However, due to the different study conditions and protocols, a direct or comprehensive comparison of iV and iS results on the remineralization of tooth enamel is difficult and cannot currently be found in the literature. To enable such a comparison, sample material and study protocols for iV and iS studies should be as similar as possible. Systematic errors can be reduced and the results of the two study types can be more comparable.

Therefore, the aim of the present study was to develop test protocols that represent a comparison between iV and iS situation. It needs to be investigated whether iV studies can reliably mimic the clinical situation, i.e., whether the remineralization behavior of human enamel when using toothpastes in an iV model under standardized conditions shows comparable results to iS procedures. The study was designed so that parameters such as tooth samples, lesion formation and test products were identical for both parts, but there were also specific parameters that deliberately differed (e.g., toothpaste application with brushes iS and without brushes iV) to keep the iV and iS (clinical) character. In this initial approach, an established iV remineralization protocol^15^ was selected and compared with a protocol mimicking the situation of the daily dental care (iS) without adaptation.

## Methods

### Study design

**Fig. 1:**
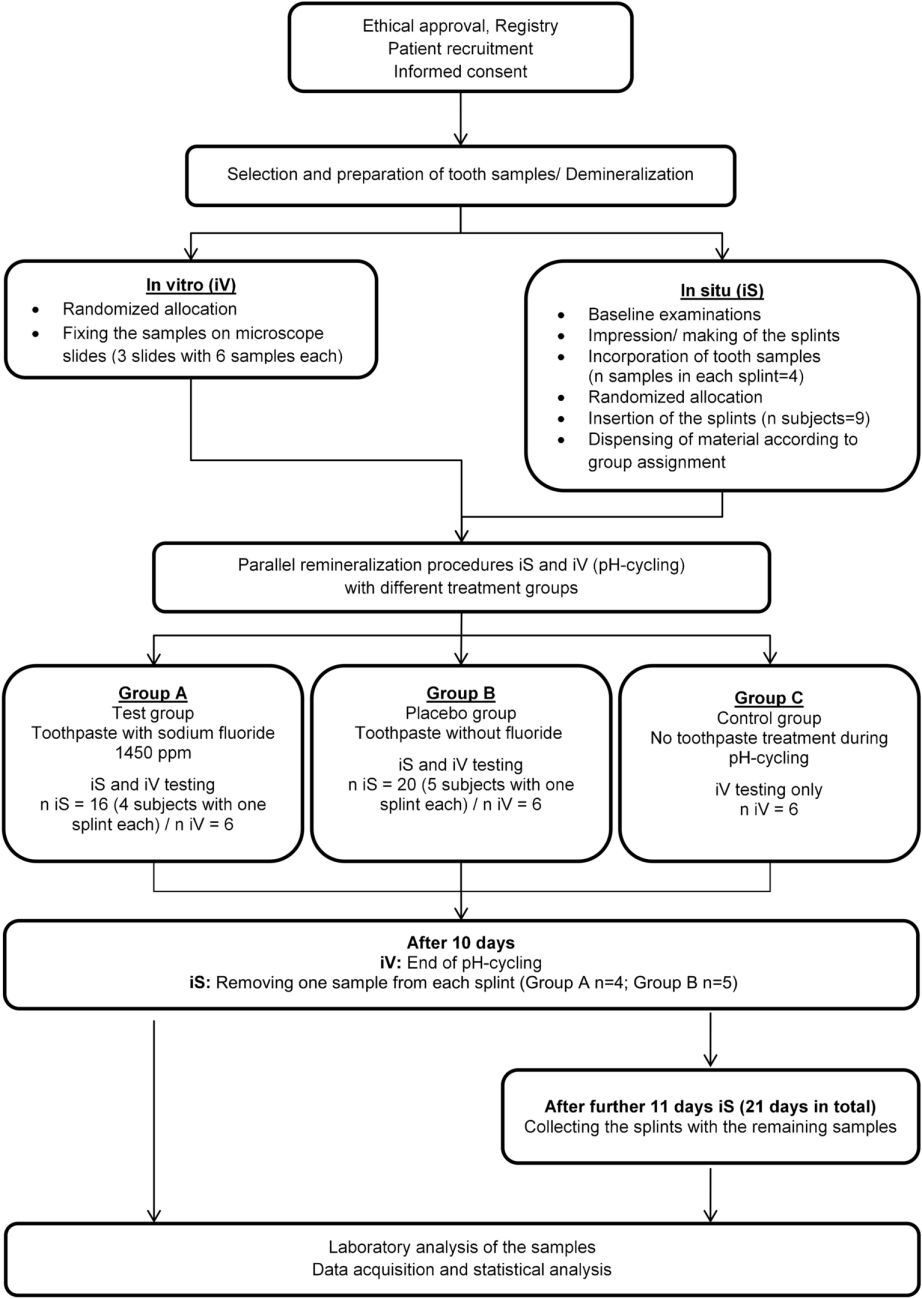
Flow diagram of the study design.

This prospective, interventional study was conducted ethically in accordance with the Word Medical Association Declaration of Helsinki. The study protocol was reviewed and approved by the Ethics Committee of the Medical Faculty of the Philipps-University of Marburg, Germany (approval number 68/22, date of approval: 09 June 2022). The study protocol was registered in the German Clinical Trials Register (DRKS00029718, date of registry: 20 July 2022). The study was conducted in two arms: in vitro and in situ. A diagram of the study design is shown in Fig. 1.

### Sample size calculation

A sample size calculation was performed prior to starting the study (program PASS 2020, v20.0.5) for the iS part of the study. The estimation of the expected effect size was based on data from previous studies (unpublished data), where a similar approach to our study was used under iV conditions. Assuming a difference of mean of 10 and the standard deviation of 7 in relation to the Δgray value results in each group, a number of 3 patients per group and 4 samples per splint (Power 0.903, α = 0.05) was calculated for the comparison of 2 groups. In order to realize the required number of cases per group even in case of dropout of one subject per group, 4 subjects per group were planned to be included. A cluster effect, which results from the fact that teeth belonging to the same patient are not independent of each other, was taken into account. In total, 4 subjects with 4 samples each were calculated per group, indicating 16 samples per group.

Since the assumed dependency between teeth on the same splint does not exist in the iV setting and hence no cluster effect occurs, the 6 samples in each group was presumed as statistically sufficient in the laboratory based setting (based on preliminary unpublished data).

### Samples for in vitro and in situ use

Extracted human third molars were used for preparation of the samples. The teeth were stored in a 0.001% sodium azide solution after extraction for disinfection for 10 days and were cleaned afterwards using scalers and brushes (miniature tooth cleaning brushes, Pluradent, Offenbach, Germany). Teeth with visible enamel defects or carious lesions were excluded from the study. Then the teeth were stored in deionized water to protect the mineral content and were additionally sterilized (Co-60 gamma irradiation) (BBF Sterilisationsservice GmbH, Kernen, Germany). The teeth were kept in deionized water until sample preparation for the study.

### Preparation of the samples

**Fig. 2:**
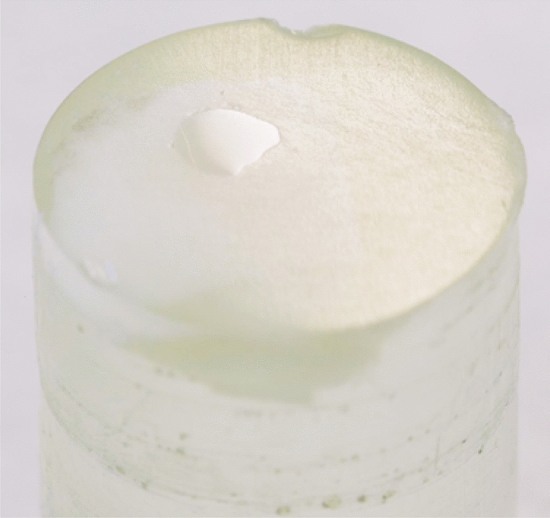
Enamel sample (whitish slab) embedded in clear epoxy resin.

The crown of the teeth was sectioned with a diamond-bladed saw (Minitom, Struers GmbH, Germany) to 7 enamel specimens each. The enamel specimens were embedded in epoxy resin (EpoFix, Struers GmbH, Germany) and ground (Minitech 333, SA Presi, France) with silicon carbide paper (120–4000 grit, Struers GmbH, Germany). The prepared samples had a diameter of 9 mm and a height of 2 mm. The area of the exposed enamel varied approximately between 4 and 8 mm^2^ (Fig. 2). After generation of artificial demineralization, the samples were randomly assigned to iV and iS remineralization group. In vitro samples were half-covered with removable two component silicon glue (Reprorubber Thin Pour, Flexbar Machine Corporation, USA) to obtain a none remineralized reference area and were divided into three treatment groups with 6 samples each. In situ samples were half-covered with light curing adhesive (Technovit 7230 VLC, Kulzer GmbH, Germany) and were divided into two treatment groups with 16 samples each.

### Demineralization of the samples

All samples were exposed without any coverage, i.e., the whole enamel sample, to a methylcellulose/lactic acid (0.1 M, pH 4.6) model described by ten Cate et al.^16^ for 14 days at 37 °C to create an artificial carious lesion of approximately 60–80 µm.

### In vitro (iV) arm of the study

The in vitro remineralization included 3 treatment groups (A: Toothpaste 1450 ppm F- of sodium fluoride, B: Toothpaste of 0 ppm fluoride, C: No toothpaste treatment). The enamel samples were treated in a pH-cycling model for 10 days. Alternating, the samples were immersed into a demineralization solution described by ten Cate et al.^15^ for 15 min under standardized stirring (375 rpm, AM4, VELP Scientifica, Italy), rinsed with deionized water for 1 min subsequently and stored in a remineralization solution^15^ for one hour under standardized stirring (375 rpm). This cycle was conducted 5 times a day for 10 days. After the 1st and 5th demineralization, respectively, a toothpaste treatment for test groups A and B was included. The enamel samples were exposed to the toothpastes as slurries (1 part toothpaste, 2 parts remineralization solution w/w) for 6 min under gentle agitation (100 rpm, PSU-10i, LTF Labortechnik GmbH & Co. KG, Germany) without brushing. For test group C, no toothpaste treatment was conducted.

### In situ (iS) arm of the study

#### Volunteers

Volunteers were recruited at a dental practice (Lollar, Germany) in September 2022 and the study was conducted between October and November 2022. Nine healthy adults (7 females, 2 male) participated in the randomized, controlled and blinded iS part of the study. Four participants were assigned to group A and 5 participants to group B. The mean age of the participants was 33.8 years (22–63 years). Written informed consent was obtained prior to any study-related procedures. All volunteers lived in an area with up to 0.25 mg F−/L in the tap water, which has been constant for many years. The participants needed to be willing and able to understand all study-related procedures and follow instructions.

Inclusion criteria were:

- age ≥ 18 years,
- low caries risk (based on the CariesCare International Criteria^17^),
- normal stimulated physiological salivary flow rate (> 1 ml/min).

Exclusion criteria were patients with:

- high caries risk (based on the CariesCare International Criteria^17^,
- removable dentures in the lower jaw,
- multibracket appliance,
- recent dental surgery,
- known allergies/hypersensitivities to the materials used,
- a currently present periodontal disease,
- signs of tooth erosion (excessive drinking of acidic beverages, reflux),
- diseases or concomitant medication that impair salivary flow or cause a dry mouth.

Further exclusion criteria were:

- pregnant women,
- breastfeeding women,
- patients who were under antibiotic treatment,
- patients who refused the use of fluoride toothpaste,
- patients who refused short-term use of fluoride-free toothpaste.

**Fig. 3:**
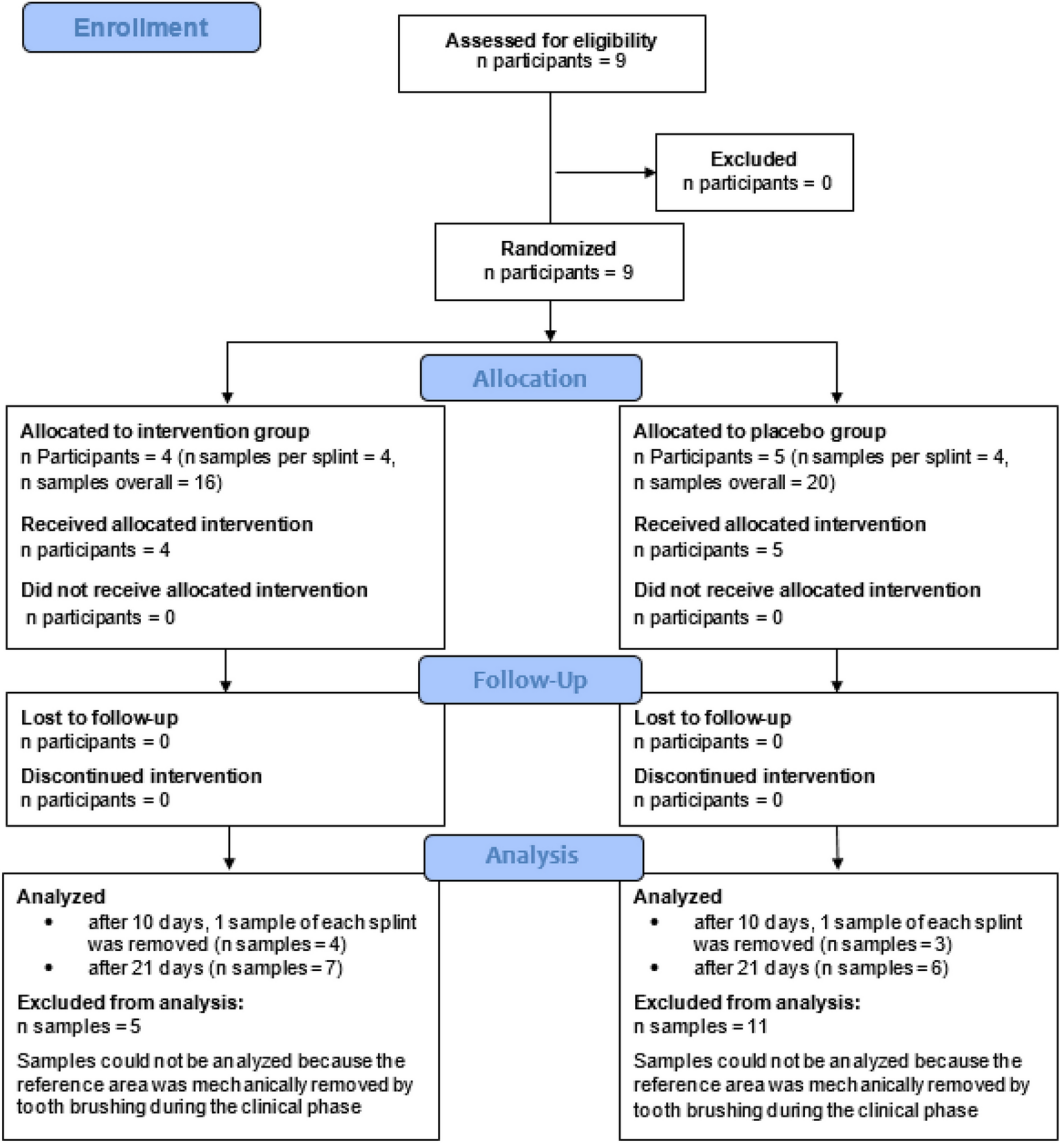
Consort diagram.

The consort diagram for the clinical part is displayed in Fig. 3.

### Preparation of the study and appliances

After the baseline examination, participants were randomly assigned to one of the two groups (group A or B) according to a simple randomization list. Group A used a fluoride-containing toothpaste (1450 ppm F- of sodium fluoride), group B a fluoride-free one (0 ppm F-). Applied toothpastes were identic to the ones used for in vitro remineralization. The list was created with MedCalc Statistical Software, v20.010 (Ostend, Belgium, www.medcalc.org) and was stored at a location with a person not involved in the study. Once a subject consented to participate in the study, the group assignment was requested by telephone via this office, which transmitted the information about the group assignment to the investigator by e-mail. The toothpastes were also dispensed via this central office. Both the investigator and the subject were blinded.

Both groups received toothpastes filled in neutral tubes. The toothpastes were specially produced for the study (DENTAL-Kosmetik GmbH Co. KG, Dresden, Germany). Furthermore, each participant received two soft toothbrushes (Dr. Best Classic, Haleon, Munich, Germany), one for the lead-in phase and one for the main study part, respectively. For the lead-in phase, each test person was given a fluoride-free toothpaste to use for dental care for seven days. Precise wearing and cleaning instructions were explained to the test subjects and given to them in writing. They were also given a record sheet to document daily use.

**Fig. 4:**
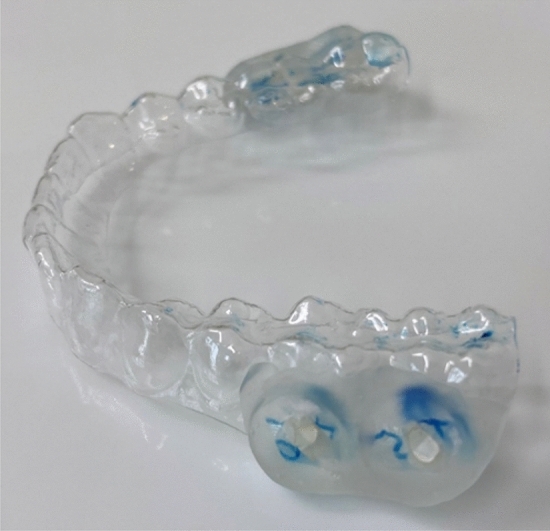
Splint with the attached specimen. The blue marks are the labels on the back of the sample for exact allocation.

From each participant mandible and maxilla impressions were taken and individual removable acrylic appliances (0.5 mm) were prepared (Erkodur clear, Erkodent, Pfalzgrafenweiler, Germany). Two specimens were fixed on each buccal flange of the appliance (Paladur Powder and Liquid, Kulzer, Hanau, Germany) (Fig. 4). Care was taken to avoid maxillary teeth coming in contact with the specimens.

### Treatment during in situ phase

The participants were instructed to brush their teeth using the fluoride-free toothpaste for seven days (wash-out phase) in order to minimize the amount of fluoride in the oral reservoirs for standardization of the initial study conditions as far as possible. Afterwards, subjects were wearing dental appliances with samples for 21 days, 21–22 h each day. The appliances were taken out for taking meals and should be kept moist in plastic boxes which were handed out to the participants. The splints should immediately be reinserted into the mouth afterwards. These times were noted and added at the end of every day to ensure similar wearing times for each test person. After the 7-day wash-out phase, each subject was given a fluoride toothpaste (group A) or a fluoride-free toothpaste (group B) according to the group allocation. Subjects brushed both their teeth and the splint separately twice a day for 2 min with the same toothbrush and toothpaste. No other dental products (mouth rinse, gels, etc.) except dental floss were to be used throughout the study. Volunteers were instructed to follow their normal eating habits throughout the study as far as possible.

After 10 days, one sample per participant was removed to obtain results from this time point (corresponding to 10 days in the in vitro part).

At the end of the total treatment phase (21 days after start of wearing the appliances), all samples were removed from the appliance. All samples from 10 and 21 days time point were rinsed with dH_2_O and disinfected and analyzed by µCT measurements.

### Analysis of the specimens

**Fig. 5:**
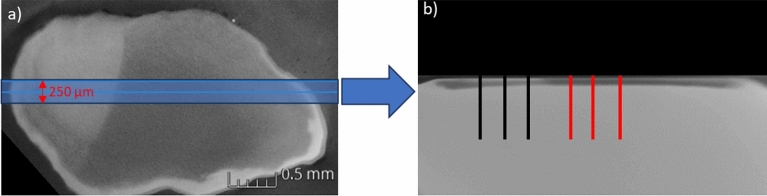
Generation of a digital cross section from µCT 3D data set. A sample range of interest (250 µm) was chosen from the surface view (**a**). Cross section image (**b**) depicts mean gray values of each voxel within the defined range of 250 µm width. Three line profiles each for demineralized (red) and remineralized (black) region were generated.

After 10d pH-cycling for the iV samples and 10d/21d treatment for the iS samples, samples were scanned without any further preparation using µCT (Nanomex 180NF, Phoenix). All samples were recorded under standardized conditions (75 kV, 160 µA). To avoid artefacts as beam hardening effect on edge region or interface or minimize their impact, surface sides of two samples, respectively, were fixed with removable two component silicon (Reprorubber Thin Pour, Flexbar Machine Corporation, USA) glue and were investigated simultaneously. From the obtained 3D data, digital cross section images were generated by averaging the respective gray values per pixel in a range of 250 µm (Fig. 5). The gray values correlate with the density of the enamel, i.e., the higher the gray value, the higher the mineral density.

**Fig. 6:**
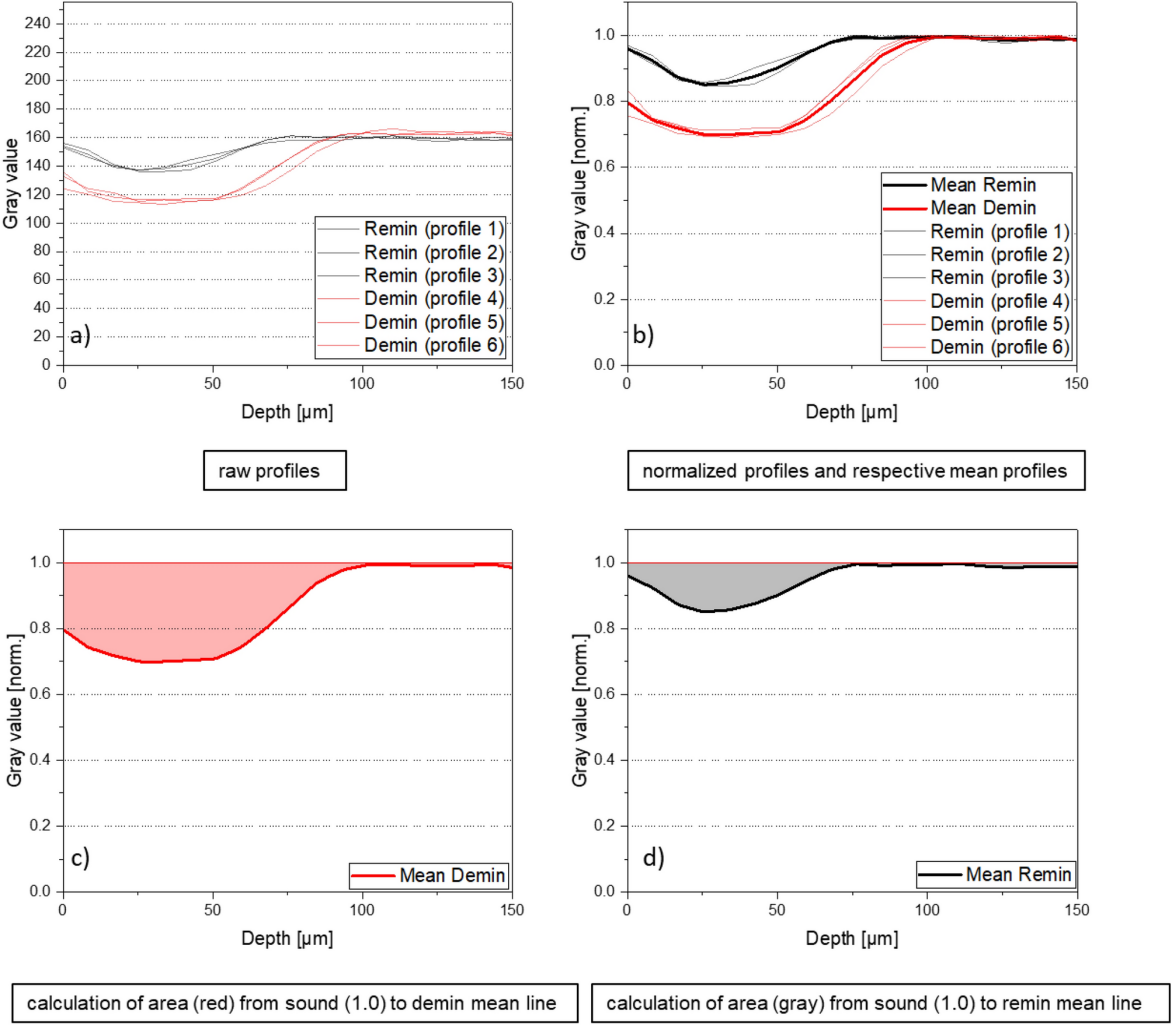
Raw profiles (**a**), normalized and averaged profiles (**b**) and the mean gray value profiles for demineralized (**c**, red) and remineralized (**d**, black) region. Area A (ΔZ) is calculated from sound to demin (light red) and remin (gray) mean line, respectively.

The evaluation of the mineral gain or percentage remineralization was performed in the form of a relative gray value comparison of de- and remineralized regions within one enamel sample. Therefore, 3 grayscale value line profiles were taken from de- and remineralized regions of each sample, averaged and normalized to sound enamel. The line profiles were taken randomly over the respective region excluding the direct edge areas, i.e., transition to embedding material. The normalization was conducted for each single profile as maximum normalization, whereas each data point from the profile was divided by the maximum gray value, which is related to the sound enamel and resulting in approx. 1.0. By integration, the area A (ΔZ) from sound (1.0) to the respective mean gray value profile was determined (Fig. 6). For quantifying the remineralization effect of the test toothpastes, the relative difference of the areas (rel.ΔZ) was calculated^18^ according to:

$$rel.\Delta Z=\frac{{A}_{(demin)}-{A}_{(remin)}}{{A}_{(demin)}}\times 100\%$$

### Statistical analysis

Statistical analyses were performed using MedCalc Statistical Software, v22.021. Data were tested for normal distribution using the Shapiro-Wilk’s test (p > 0.05). T-Test for dependent and independent data and analysis of variance (ANOVA) with Tukey–Kramer post-hoc tests were used for further analyses. The significance level was set at α = 0.05.

## Results

During the in situ part no breach of protocol was recorded and no participant was excluded from analysis. No adverse reaction was reported. The splints were worn on average 21.6 h (19–23 h).

In total, 54 samples were included in the study (18 in vitro, 36 in situ). From the iS samples, 16 were not suitable for further analysis (group A = 5, group B = 11) since the remineralization area was mechanically removed by tooth brushing and wear during the clinical phase. Hence, 20 specimens of the iS part and all specimens of the iV part were available for statistical evaluation (n total = 38). Delta gray values for all samples are summarized in Tables 1 and 2.

**Table 1:** Results of the delta gray values of the in vitro part after de- and remineralization (AU: arbitrary units, SD: standard deviation). Rel.ΔZ is calculated separately for each sample, i.e., the mean value shown does not result from the respective mean ΔZ of demin and remin.

In vitro	N sample	A (ΔZ) Demin [AU] [mean ± SD]	A (ΔZ) Remin [AU] [mean ± SD]	rel.ΔZ [%] [mean ± SD]
A (test group), 10 days	6	− 28.5 ± 7.2	− 17.5 ± 8.2	40.2 ± 18.4^a^
B (placebo group), 10 days	6	− 21.7 ± 6.9	− 22.8 ± 7.5	− 6.2 ± 19.0^a,b^
C (control group), 10 days	6	− 22.5 ± 8.4	− 23.3 ± 8.4	− 3.7 ± 12.5^a,b^

Different superscript letters indicate different p-values: a: p < 0.001; b: p = 0.793.

**Table 2:** Results of the delta gray values of the in situ part after de- and remineralization (AU: arbitrary units, SD: standard deviation). Rel.ΔZ is calculated separately for each sample, i.e., the mean value shown does not result from the respective mean ΔZ of demin and remin.

**In situ**	N sample	A (ΔZ) Demin [AU] [mean ± SD]	A (ΔZ) Remin [AU] [mean ± SD]	rel.ΔZ [%] [mean ± SD]
A (test group), 10 days	4	− 32.0 ± 6.4	− 27.4 ± 0.9	11.5 ± 18.8^a,c^
A (test group), 21 days	7	− 25.8 ± 9.4	− 14.0 ± 6.4	46.1 ± 13.6^a,b^
B (placebo group), 10 days	3	− 28.5 ± 9.0	− 20.8 ± 6.2	25.2 ± 16.9^c^
B (placebo group), 21 days	6	− 30.6 ± 9.1	− 26.4 ± 7.4	11.0 ± 30.2^b^

Different superscript letters indicate different p-values: a: p = 0.022; b: p = 0.034; c: p = 0.40.

The areas of the normalized gray values after demineralization were comparable for the samples iV and iS (p = 0.083), indicating same baseline conditions at the start of the study.

### Results of the in vitro samples

Mean rel.ΔZ of the fluoride treated samples (group A) iV was 40.2% after 10d. In comparison, the rel.ΔZ in the fluoride-free (B) and negative control group (C) was significantly lower (Table 1, p < 0.001). Both reference test groups (placebo and control) did not differ significantly (p = 0.793).

### Results of the in situ samples

In the iS part of the study, rel.ΔZ in group A was 11.5% after 10 days and 46.1% after 21 days (Table 2, p = 0.022). Rel.ΔZ in samples from group B (placebo group) were 25.2% after 10d and 11.0% after 21d (p = 0.80). The comparison of groups A and B revealed significant difference in rel.ΔZ only after 21 days (p = 0.034), but not after 10d (p = 0.40) as in the iV part of the study.

### Comparison of results between in vitro and in situ samples

The comparison between iV and iS data (Tables 1 and 2) showed no significant differences for group A (test group), neither after 10d nor after 21d iS (p = 0.055 and p = 0.532, respectively), whereas rel.ΔZ for 21d iS was more clearly in the range of iV 10d. The difference between iV and iS was not statistically significant in group B (placebo group) either (10d iS: p = 0.054 and 21d iS: p = 0.272). In general, higher remineralization was detected for iS samples in the test group.

## Discussion

In the present study, the extent to which re- and demineralization processes of the oral environment can be mimicked by in vitro test methods was evaluated compared to an in situ model. It was shown that the remineralization efficacy of fluoridated toothpastes was quantitatively comparable for iV and iS treated samples. The remineralization capability of both toothpastes could be differentiated significantly for both procedures (in situ p = 0.034, in vitro p = 0.001), i.e., the superiority of fluoride-containing toothpaste over fluoride-free toothpaste under similar remineralization conditions has been demonstrated.

However, this differentiation was observed for the iS setting only after 21 days but not after 10 days which were used in vitro. Remineralization for both iS test groups after 10 days can be related to the presence of oral fluoride reservoirs or the Ca and P content in the saliva of the test subjects. In particular for the placebo group, the relatively high remineralization effect could be explained by potential fluoride depots in the saliva and on the tooth surface, especially in the colonizing plaque^19^ from which fluoride is gradually released. This leads generally to higher remineralization and lower demineralization. Other studies have shown that even lowest concentrations of fluoride promote remineralization and inhibit demineralization of enamel and dentin^20^. Fluoride intake via food and drinking tap water in combination with existing fluoride reservoirs is probably sufficient to remineralize demineralized areas in the short term, despite the use of a fluoride-free toothpaste. The artificial lesions in the current study showed the same mineral deposition over them at the first 10 days, irrespective of whether the tooth slabs were brushed with or without fluoridated toothpaste. The accumulation of fluorides (depot effect) reflects the need of wash-out phase to be implemented to standardize the evaluation procedures. During this period, systemically stored fluorides are eliminated and no additional fluorides are added. This can reduce systematic errors. An average value of 6.3 days for the wash-out phase determined in a selection of 27 studies confirms the value of 7 days in this study^21^. The one-week washout period in the present study was in line with previous studies^22,23^. In the present study the stagnating or rather decreasing (not significantly) remineralization of the placebo group after 21 days (11.0%) compared to 10 days (25.2%) suggests an even higher level of fluoride in oral reservoirs. Duckworth et al.^19^ also show that the fluoride concentration in saliva only returns to the original baseline value after about 2 weeks. This may indicate that the wash-out phase should last longer than 7 days in future studies. However, this cannot always be carried out due to patient cooperation since the use of fluoride toothpaste is recommended for daily caries prevention. In addition, a determination of fluoride concentrations in plaque and saliva before the start of the study is recommended. It would also be of interest to observe, whether the remineralization values would continue to decrease or stagnate in the placebo group with a longer study duration.

Saliva promotes remineralization iS not only due to reservoir released fluoride but also due to salivary pellicle, a thin acellular organic film. The pellicle does not have only the function of substrate protection, lubrication and hydration, but also as remineralizing agent^24^. The saliva is supersaturated with calcium and phosphate ions. By forming a surface pellicle, the acid-induced dissolution rate can be slowed down by promoting the continuous precipitation of calcium phosphates and selective ion exchange^25,26^. This is another explanation why the iS remineralization value is similar in both the fluoride-containing and fluoride-free group at the beginning (after 10 days).

The other way around, fluoride reservoirs need several weeks to fill up and thus reach their full capacity. In addition, the oral fluoride retention is influenced by numerous factors such as tooth structure, interdental spaces and the surface coating of hard and soft tissues^27^. This explains why the relative ΔZ after 21 days (46.1%) in group A iS was more than three times higher compared to 10 days (11.5%) (Table 1), and 10d iS was lower than detected for iV (40.2%). In order to achieve even better comparability in further studies, samples should also be analyzed after an even longer study period and at shorter intervals. In this way, more precise statements can be made about the amount of fluoride in oral reservoirs in order to be able to transfer this even better to iV situations. Selected iS studies may be replaced by iV studies in the future, when a shorter time period for the study procedure is needed or the selection of appropriate participants is not possible. The fact that similar remineralization to iS after 21d could be observed iV after only 10 days shows that processes iV presumably take place faster and can be influenced more precisely.

Different protocols are existing to conduct iV or iS studies to evaluate remineralization of enamel surface. The protocols show numerous variations and generally, comparison is only possible to a certain degree. Among iV study methods, pH-cycling models have proven to be particularly suitable for the investigation of de- and remineralization^12,28–30^. In such models, a combination of demineralization and remineralization solutions mimics the dynamics of mineral loss and gain during caries formation in the enamel^31^. It is intended to mimic the dynamics of oral flora and make iS and iV studies comparable. The use of tooth samples from the same tooth for both iS and iV is crucial for the comparability of the results. The reason for this is the highly variable composition of the teeth, caused by genetic influences, environmental conditions and age. This may lead to large differences in their reaction to demineralization and remineralization^32^. In our study, this limitation was addressed by assigning the samples from each tooth to the same group iV and iS.

A common appliance type reported in iS studies is the palatal device as the carrier of dental substrates^21^, but there are also studies using appliances in the mandible^12,21^. A possible reason for wearing splints in the maxilla is the result of a study by Zero et al.^27^, which showed an intermediate accumulation of fluorides in the upper posterior maxillary sulcus compared to other soft tissue zones. In the lower posterior buccal sulcus, the accumulation was slightly increased. To standardize this influence of fluoride storage in soft tissue zones, maxillary splints are probably chosen more often. In our study we did not evaluate the samples at short intervals but after 10 or 21 days. Hence the different short-term storage capacity in the soft tissue during the first 24 h would play a subordinate role.

Furthermore, the recycling of fluoride in the saliva of the parotid gland as a result of accidental ingestion of home fluoride products does not appear to make a clinically significant contribution to oral fluoride levels. This emphasizes that both mandibular and maxillary splints are possible to use^33^.

The decisive factor for the choice of a mandibular splint, the shape and material, as well as the position of the samples used in this study was the successful use in a previous iS study by the same working group^23^. The wearing and speaking comfort was well accepted by the participants. Fabrication was relatively simple and inexpensive. We therefore decided to use this type of splint again in the mandible. In iS models, the oral hygiene of the individual test subjects plays a decisive role. Various factors can influence remineralization. Thorough oral health instructions during the study period can lead to altered results even before the start of the study and during the study phase^34^. However, brushing rituals rarely follow a protocol in everyday life and do not always correspond to the norm. Another variability factor is the rinsing of the mouth after brushing the teeth. By rinsing the mouth for a shorter time more fluoride remains in the oral cavity^35^. A recently published study shows that the mouth should not be rinsed after spitting out the toothpaste. In this way, more effective remineralization can be achieved by fluorides^3^. Since the amount of toothpaste used also influences remineralization, studies should precisely regulate the amount of toothpaste to be applied^36^. In our study, the amount of toothpaste used by the participants varied from 15 to 83 g in the study phase over 21 days. Furthermore, the dilution degree of the toothpastes highly depends on the saliva (flow) and can vary. In the in vitro part, a standardized amount of 15 g toothpaste as well as a standardized dilution degree (1:2 toothpaste vs. water) was used per cycle.

The brushing technique and the force used when manually brushing the teeth is variable and often too strong. Despite the use of soft toothbrushes in our study, mechanical overload was also observed in this study. This resulted in the fact that not all samples from the iS group could be analyzed, as the cover on the demineralized area was detached and thus no reference area could be analyzed. Furthermore, on some samples, the area to be remineralized was worn, i.e., an abrasion by the brushing procedure took place and thus no assessment of the remineralization could be conducted. Interestingly, this abrasion effect was mainly observed for the placebo treated samples. This could be a hint for the better remineralization of the fluoride treated samples resulting in a re-hardening of the enamel and thus an abrasion protecting effect. Such limitations can be reduced by using a different coverage material for the control side, for example. However, attention must be paid here to biocompatibility. Another possibility would be to analyze all samples after demineralization with the Micro CT and then embed them in the splints without further covering and analyze them again after remineralization. However, this leads to longer and more expensive analysis times. The design of the appliance could have also played a crucial rule in such abrasion. Maybe if the samples were fixed in the appliances and surrounded by troughs, such abrasion would not have occurred. This was not an issue in the iV setting, as the standardized toothpaste treatment was conducted by only storing in the toothpaste slurry with no brushing step to reveal the pure chemical interaction of the respective toothpastes with the enamel/lesion. This shows another limitation when conducting iS studies. It is worth considering whether the approach used in other studies of only distributing the toothpaste foam over the samples and not mechanically cleaning the samples themselves is more appropriate^37^.

## Conclusion

The results of the present study propose that outcomes on remineralization potential of toothpastes with and without fluorides were comparable iS and iV. A similar remineralization was obtained iV compared to iS after less time (10 days vs. 21 days). The data suggests that selected in situ studies can be replaced by in vitro studies in the future. Using pH-cycling, subject-specific fluctuations can be eliminated. In the long term, pH-cycling models may replace the rather time-consuming and cost-intensive in situ protocols at least for selected studies on remineralization procedures.

## Data Availability

All data generated or analyzed during this study are included in this article. Further inquiries can be directed to the corresponding author.
